# Dynamic immune reconstitution and clinical outcomes in different chimerism statuses of HLA-matched transplantation for severe aplastic anemia

**DOI:** 10.1093/stcltm/szag009

**Published:** 2026-03-27

**Authors:** Ming-Hao Lin, Zheng-Li Xu, Ying-Jun Chang, Hui-Dong Guo, Lan-Ping Xu, Yu Wang, Xiao-Hui Zhang, Yi-Fei Cheng, Yuan-Yuan Zhang, Xiao-Dong Mo, Yu-Qian Sun, Ting-Ting Han, Jing-Zhi Wang, Yao Chen, Yu-Hong Chen, Huan Chen, Wei Han, Xiao-Jun Huang

**Affiliations:** Peking University People’s Hospital, Peking University Institute of Hematology, National Clinical Research Center for Hematologic Disease, Beijing Key Laboratory of Cell and Gene Therapy for Hematologic Malignancies, Peking University, Beijing 100044, China; Peking University People’s Hospital, Peking University Institute of Hematology, National Clinical Research Center for Hematologic Disease, Beijing Key Laboratory of Cell and Gene Therapy for Hematologic Malignancies, Peking University, Beijing 100044, China; Peking University People’s Hospital, Peking University Institute of Hematology, National Clinical Research Center for Hematologic Disease, Beijing Key Laboratory of Cell and Gene Therapy for Hematologic Malignancies, Peking University, Beijing 100044, China; Peking University People’s Hospital, Peking University Institute of Hematology, National Clinical Research Center for Hematologic Disease, Beijing Key Laboratory of Cell and Gene Therapy for Hematologic Malignancies, Peking University, Beijing 100044, China; Peking University People’s Hospital, Peking University Institute of Hematology, National Clinical Research Center for Hematologic Disease, Beijing Key Laboratory of Cell and Gene Therapy for Hematologic Malignancies, Peking University, Beijing 100044, China; Peking University People’s Hospital, Peking University Institute of Hematology, National Clinical Research Center for Hematologic Disease, Beijing Key Laboratory of Cell and Gene Therapy for Hematologic Malignancies, Peking University, Beijing 100044, China; Peking University People’s Hospital, Peking University Institute of Hematology, National Clinical Research Center for Hematologic Disease, Beijing Key Laboratory of Cell and Gene Therapy for Hematologic Malignancies, Peking University, Beijing 100044, China; Peking University People’s Hospital, Peking University Institute of Hematology, National Clinical Research Center for Hematologic Disease, Beijing Key Laboratory of Cell and Gene Therapy for Hematologic Malignancies, Peking University, Beijing 100044, China; Peking University People’s Hospital, Peking University Institute of Hematology, National Clinical Research Center for Hematologic Disease, Beijing Key Laboratory of Cell and Gene Therapy for Hematologic Malignancies, Peking University, Beijing 100044, China; Peking University People’s Hospital, Peking University Institute of Hematology, National Clinical Research Center for Hematologic Disease, Beijing Key Laboratory of Cell and Gene Therapy for Hematologic Malignancies, Peking University, Beijing 100044, China; Peking University People’s Hospital, Peking University Institute of Hematology, National Clinical Research Center for Hematologic Disease, Beijing Key Laboratory of Cell and Gene Therapy for Hematologic Malignancies, Peking University, Beijing 100044, China; Peking University People’s Hospital, Peking University Institute of Hematology, National Clinical Research Center for Hematologic Disease, Beijing Key Laboratory of Cell and Gene Therapy for Hematologic Malignancies, Peking University, Beijing 100044, China; Peking University People’s Hospital, Peking University Institute of Hematology, National Clinical Research Center for Hematologic Disease, Beijing Key Laboratory of Cell and Gene Therapy for Hematologic Malignancies, Peking University, Beijing 100044, China; Peking University People’s Hospital, Peking University Institute of Hematology, National Clinical Research Center for Hematologic Disease, Beijing Key Laboratory of Cell and Gene Therapy for Hematologic Malignancies, Peking University, Beijing 100044, China; Peking University People’s Hospital, Peking University Institute of Hematology, National Clinical Research Center for Hematologic Disease, Beijing Key Laboratory of Cell and Gene Therapy for Hematologic Malignancies, Peking University, Beijing 100044, China; Peking University People’s Hospital, Peking University Institute of Hematology, National Clinical Research Center for Hematologic Disease, Beijing Key Laboratory of Cell and Gene Therapy for Hematologic Malignancies, Peking University, Beijing 100044, China; Peking University People’s Hospital, Peking University Institute of Hematology, National Clinical Research Center for Hematologic Disease, Beijing Key Laboratory of Cell and Gene Therapy for Hematologic Malignancies, Peking University, Beijing 100044, China; Peking University People’s Hospital, Peking University Institute of Hematology, National Clinical Research Center for Hematologic Disease, Beijing Key Laboratory of Cell and Gene Therapy for Hematologic Malignancies, Peking University, Beijing 100044, China; Peking-Tsinghua Center for Life Sciences, Academy for Advanced Interdisciplinary Studies, Peking University, Beijing 100871, China

**Keywords:** immune reconstitution, mixed chimerism, full donor chimerism, HLA-matched transplantation, severe aplastic anemia

## Abstract

This retrospective study examines the clinical outcomes and immune reconstitution dynamics in patients with severe aplastic anemia (SAA) exhibiting mixed chimerism (MC) compared with those with full donor chimerism (FDC) following HLA-matched hematopoietic stem cell transplantation (HSCT). Analysis of propensity score-matched cohorts (23 MC vs 69 FDC) revealed comparable 5-year overall survival (OS: 87.0% vs 92.8%, *P* = .433) but significantly inferior failure-free survival (FFS: 47.8% vs 87.0%, *P* < .001) in patients with MC due to a higher incidence of graft failure (52.2% vs 8.7%, *P* < .001). Longitudinal immune profiling revealed delayed recovery of myeloid and lymphoid lineages in patients with MC at 12 months post-HSCT, with pronounced deficits in adaptive immunity. Specifically, CD8^+^CD28^+^ T cell counts were consistently reduced at 1 month (median, 20 vs 41 cells/μL, *P* = .049), 3 months (median, 86 vs 153 cells/μL, *P* = .024), and 6 months (median, 109 vs 160 cells/μL, *P* = .001), and CD4^+^CD25^+^ T cells were diminished at 6 months (median, 11 vs 20 cells/μL, *P* = .006). Multivariate analysis revealed that elevated CD8^+^CD28^+^ T cell levels at 3 months (≥140 cells/μL) were an independent predictor of improved FFS (HR = 0.30, *P* = .035). These findings highlight MC-associated immune dysregulation, particularly impaired CD28-costimulated T cell and CD4^+^CD25^+^ T cell reconstitution, as a key mediator of graft instability. This study underscores the prognostic value of early immune monitoring and suggests therapeutic strategies that target T cell recovery to mitigate MC-related risk in patients with SAA.

## Graphical abstract

**Graphical Abstract:**
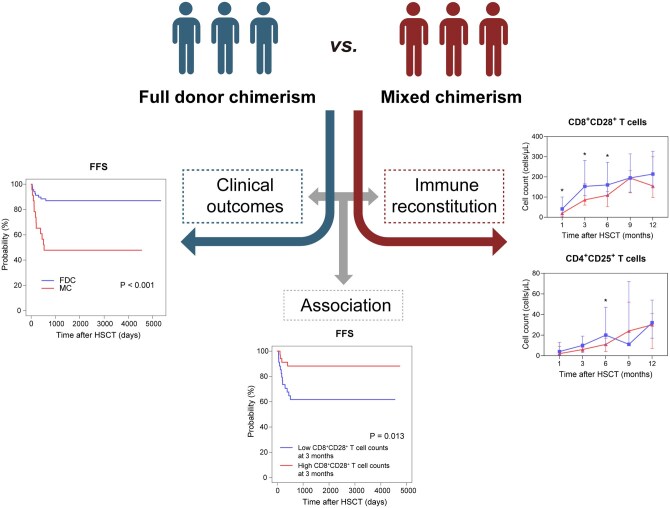

Significance statement Severe aplastic anemia is a life-threatening blood disorder often treated with hematopoietic stem cell transplantation. While achieving full donor chimerism is ideal, mixed chimerism remains a clinical challenge with unclear immune reconstitution in HLA-matched transplantation. This study reveals that mixed chimerism is associated with poorer failure-free survival, and is linked to delayed immune recovery, including CD8^+^CD28^+^ T cells and CD4^+^CD25^+^ T cells. By highlighting the role of specific immune cell populations, the research underscores the importance of early immune monitoring and suggests targeted strategies to enhance immune reconstitution, potentially improving long-term outcomes.

## Introduction

Severe aplastic anemia (SAA), a life-threatening bone marrow (BM) failure syndrome, has increasingly been treated with allogeneic hematopoietic stem cell transplantation (allo-HSCT) as a curative approach.^1^^,^^2^ Recent advancements in conditioning regimens and supportive care have facilitated successful outcomes across various donor types, with 5-year survival rates reaching 80-90% in HLA-matched related and unrelated donor (MRD/MUD) HSCT.^3^^,^^4^ The stable engraftment of hematopoietic stem cells within the recipient is a critical determinant of transplantation success. Following transplantation, donor-derived hematopoietic stem cells gradually engraft and replace the recipient’s original blood cells, a process referred to as chimerism, which is categorized into full donor chimerism (FDC), mixed chimerism (MC), and graft rejection.^5^ Although achieving FDC is a therapeutic objective, MC, defined by the persistent coexistence of donor and recipient hematopoietic cells (5-95% donor-derived cells), remains challenging to prevent in clinical settings. HLA-matched HSCT rather than haploidentical (haplo-) HSCT poses a risk factor for MC, which is observed in 26-55% of patients with SAA undergoing MRD/MUD HSCT.^6–10^ Prior studies have focused predominantly on the clinical implications and risk factors associated with MC, highlighting its correlation with suboptimal clinical outcomes, especially graft failure (GF). However, these investigations largely overlooked immune reconstitution patterns, which could play a pivotal role in mediating graft stability and influencing clinical outcomes.

This retrospective study seeks to address these gaps by systematically comparing clinical outcomes and longitudinal immune reconstitution between the MC and FDC cohorts in patients with SAA undergoing HLA-matched transplantation. By integrating clinical and immunologic datasets, this study aims to elucidate the mechanisms that connect chimerism status to transplant success, thereby informing risk stratification and guiding targeted interventions.

## Methods

### Patients

This retrospective study included 23 patients diagnosed with SAA who developed MC after their initial HLA-matched allo-HSCT at Peking University People’s Hospital between January 2010 and December 2022. Eligibility criteria required patients to have available immune reconstitution data at one or more predefined intervals. A control cohort consisting of 69 patients exhibiting FDC was established through propensity score matching (PSM) to minimize selection bias to compare clinical outcomes and immune reconstitution. AA was diagnosed according to the Camitta criteria.^11^ The last follow-up for all surviving patients was January 1, 2025. The study protocol received approval from the Ethics Committee of Peking University People’s Hospital (2023PHB321-001) and was conducted in compliance with the Declaration of Helsinki. Informed consent was obtained from all participants or their guardians.

### Transplantation procedures

Four conditioning regimens were utilized for patients with SAA in this cohort. The majority (*n* = 57) received a cyclophosphamide (Cy)/antithymocyte globulin (ATG) regimen, consisting of intravenous Cy (50 mg/kg/d) and ATG (2.5 mg/kg/d) administered over a four-day period (Days −5 to −2). A subset of patients (*n* = 19) underwent a busulfan (Bu)/Cy/ATG regimen, which included intravenous Bu (3.2 mg/kg on Day −8) followed by Cy (50 mg/kg/d) and ATG (2.5 mg/kg/d) once daily on Days −5 to −2. For patients requiring reduced toxicity, a Bu/fludarabine (Flu)/Cy/ATG regimen (*n* = 11) comprising Bu (3.2 mg/kg on Day −8), Flu (30 mg/m^2^/d on Days −6 to −2), Cy (25 mg/kg/d on Days −5 to −2), and ATG (2.5 mg/kg/d on Days −5 to −2) was employed. Additionally, the Cy/Flu/ATG regimen was administered to a smaller group (*n* = 5), which received Flu (30 mg/m^2^/d on Days −6 to −2) with Cy (50 mg/kg/d) and ATG (2.5 mg/kg/d) on Days −5 to −2. Regimens containing flu were primarily selected based on individual patient risk stratification, specifically for those with high cardiac toxicity risk or a hematopoietic cell transplantation comorbidity index ≥3. In contrast, conditioning regimens incorporating Bu have been applied to a subset of patients since 2019.^12^ All patients received standardized graft-versus-host disease (GvHD) prophylaxis with cyclosporine A (CsA), mycophenolate mofetil (MMF), and short-term methotrexate (MTX). Specifically, CsA was initiated at a dosage of 2.5 mg/kg/d starting on Day −3 and was delivered via intravenous infusion until the patient’s intestinal function normalized, at which point the administration was transitioned to oral delivery. A gradual tapering of CsA was scheduled to commence one year following transplantation, with the aim of complete cessation thereafter. MMF was administered orally at a dosage of 1 g/d for adults and 0.5 g/d for pediatric patients, initiated concurrently with CsA, and was tapered upon successful engraftment. MTX was administered intravenously at a dosage of 15 mg/m^2^ on Day +1, followed by 10 mg/m^2^ on Days +3 and +6 for MRD, or on Days +3, +5, and +11 for MUD post-transplantation. All stem cell grafts from MRD/MUD were infused fresh without cryopreservation. Detailed descriptions of stem cell collection and supportive care protocols have been previously described.^12^

### Chimerism analysis and definitions

**Figure 1.**
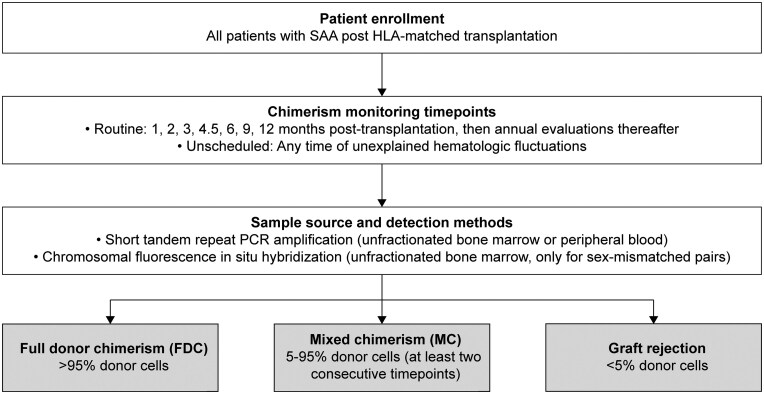
Flowchart for chimerism analysis and classification in patients with SAA following HLA-matched transplantation. Chimerism assessments were routinely conducted at 1, 2, 3, 4.5, 6, 9, and 12 months post-transplantation, with annual evaluations thereafter, alongside unscheduled tests in cases of unexplained hematologic fluctuations. The analysis of chimerism was performed using unfractionated BM or PB samples to determine the proportion of donor-derived hematopoietic cells. This was validated through short tandem repeat PCR for both BM and PB samples, with optional chromosomal fluorescence in situ hybridization employed exclusively for sex-mismatched donor-recipient pairs. Chimerism classification was defined as follows: FDC was characterized by the presence of more than 95% donor-derived cells; MC was confirmed when donor-derived cells constituted 5-95% and this proportion was consistent across two or more consecutive assessments; a donor-derived cell proportion of less than 5% was further classified as graft rejection.

The flowchart of chimerism analysis was presented in Figure 1. Chimerism analysis was conducted using unfractionated samples from the recipient’s BM or peripheral blood (PB) to thoroughly assess the proportion of donor-derived hematopoietic cells. Evaluations were carried out at 1, 2, 3, 4.5, 6, 9, and 12 months post-transplantation, followed by annual assessments. In addition, chimerism analyses were performed in response to any unexplained hematologic fluctuations at any time. The analyses were validated through short tandem repeat PCR amplification using BM or PB samples and can also be confirmed by chromosomal fluorescence in situ hybridization using BM in case of sex-mismatched pairs. FDC was defined as the presence of more than 95% donor-derived hematopoietic cells in PB, whereas MC was characterized by the detection of 5-95% donor cells, with this proportion persisting across two or more consecutive evaluations.^5^

Neutrophil engraftment was identified by the first occurrence of 3 consecutive days with an absolute neutrophil count (ANC) of ≥0.5 × 10^9^/L, whereas platelet engraftment was defined by the maintenance of a platelet count of ≥20 × 10^9^/L for 7 days without transfusion. Acute and chronic GvHD (aGvHD/cGvHD) were staged using the modified Glucksberg scale and the National Institutes of Health criteria, respectively.^13^^,^^14^ The cytomegalovirus (CMV) and Epstein–Barr virus (EBV) viremia thresholds adhered to institutional protocols as previously reported.^15^ Overall survival (OS) was measured as the time from transplantation to death or the last follow-up. Failure-free survival (FFS) was defined as survival without GF, transfusion dependence, or mortality. GF included graft rejection and poor graft function in this study. Primary GF was defined as failure to achieve neutrophil engraftment by Day +28, while secondary graft failure was defined as a decline in hematopoietic function after initially successful engraftment, as previously described.^16^

### Immune reconstitution analysis

PB samples were collected from recipients in heparinized tubes at intervals of 1, 3, 6, 9, and 12 months post-HSCT for the purpose of immune cell subset analysis. The total ANC, monocyte, and lymphocyte counts were quantified using automated hematology analyzers. A comprehensive panel of antibody combinations was employed to identify CD3, CD4, CD8, CD19, CD25, CD28, CD45RA, and CD45RO through multiparameter flow cytometry (BD FACSSort, Becton Dickinson Biosciences, San Jose, CA, USA) using fluorescein isothiocyanate-, phycoerythrin-, allophycocyanin-, and peridinin chlorophyll protein-conjugated monoclonal antibodies as previously described.^17^^,^^18^ Data acquisition and analysis were conducted using CellQuest software (BD Biosciences). Simultaneously, serum immunoglobulin levels (IgA, IgG, and IgM) were assessed through immunonephelometry to assess humoral immune recovery.

### Statistical analysis

Propensity scores were estimated through multivariable logistic regression, with chimerism status as the dependent variable and the following pretransplant characteristics as covariates: patient age, sex, and donor type. A 1:3 nearest-neighbor matching algorithm without replacement was implemented using a caliper width set to 0.2 standard deviations of the logit-transformed propensity scores to ensure adequate comparability between groups. Continuous variables were presented as the medians and ranges or quartiles. Data comparisons were conducted using either Student’s *t*-test or the Mann–Whitney *U* test. Categorical variables were summarized as frequencies and percentages and were compared using either the chi-square test or Fisher’s exact test. OS and FFS were estimated by the Kaplan–Meier method. Competing risk analysis was employed for outcomes such as engraftment, GvHD, and viral reactivation (CMV/EBV), with death from other causes considered a competing risk. Differences among subgroups were assessed using the Fine–Gray test. Spearman’s rank correlation analysis was performed to evaluate the potential association between graft cell dose and the recovery of immune cell subsets post-HSCT. Given the absence of established clinical or biologically validated thresholds for immune cell subsets in the context of HSCT for SAA, a median split approach was utilized to dichotomize continuous immune cell counts into binary variables, thereby reducing the risk of overfitting. Univariate and multivariate analyses between clinical outcomes and immune reconstitution were conducted using the Cox regression model. Variables exhibiting a *P*-value of less than .1 in the univariate analysis were included in the multivariate analysis. A *P*-value of less than .05 was deemed statistically significant, with all reported *P*-values derived from two-sided hypothesis tests. All the data were analyzed using R software (version 4.2.2) and GraphPad Prism software (version 10.1.2).

## Results

### Patient characteristics

A total of 92 patients with SAA who underwent HLA-matched HSCT were retrospectively analyzed. Following PSM, 69 patients were allocated to the FDC group, and 23 were allocated to the MC group. The median age of the patients was 20 years (range, 4-51 years). In the MC group, the median time to develop MC was 3 months (range, 1-17 months). Baseline characteristics were generally comparable between the two groups, except for significant differences in the use of Bu-containing conditioning regimens (FDC 39.1% vs MC 13.0%, *P* = .021) and graft composition (Table 1). Notably, the grafts in the MC group presented significantly lower median values for mononuclear cells and CD34^+^ cells than those in the FDC group. The median follow-up durations for survivors were 69.5 months (range, 1.4-177.6 months) and 91.2 months (range, 10.9-172.2 months) in the FDC and MC groups, respectively.

**Table 1.** Baseline characteristics of the overall cohort and comparison between the FDC and MC groups.

Variable	Overall (*n* = 92)	FDC group (*n* = 69)	MC group (*n* = 23)	*P*-value
**Median age of patients at HSCT, years (range)**	20 (4-51)	20 (4-51)	17 (4-51)	.528
**Patient gender, *n* (%)**				1.000
** Male**	64 (69.6)	48 (69.6)	16 (69.6)	
** Female**	28 (30.4)	21 (30.4)	7 (30.4)	
**Disease severity, *n* (%)**				.753
** SAA**	77 (83.7)	57 (82.6)	20 (87.0)	
** vSAA**	15 (16.3)	12 (17.4)	3 (13.0)	
**Months from diagnosis to HSCT, *n* (%)**	18 (1-264)	24 (1-240)	11 (1-264)	.639
**Previous ATG treatment, *n* (%)**	16 (17.4)	11 (15.9)	5 (21.7)	.535
**Median pre-HSCT RBC transfusion, units (range)**	20 (0-336)	21 (0-336)	20 (0-324)	.862
**Median pre-HSCT platelet transfusion, units (range)**	15 (0-120)	15 (0-120)	15 (3-60)	.670
**Median pre-HSCT ferritin, ng/mL (range)**	1439 (26-12706)	1405 (77-12706)	2243 (26-5925)	.778
**HCT-CI score, *n* (%)**				.506
** 0**	79 (85.9)	58 (84.1)	21 (91.3)	
** ≥1**	13 (14.1)	11 (15.9)	2 (8.7)	
**Median age of donors, years (range)**	26 (7-56)	26 (7-56)	26 (8-56)	.943
**Donor-patient sex match, *n* (%)**				.507
** Male–male**	18 (19.6)	11 (16.7)	7 (30.4)	
** Male–female**	20 (21.7)	15 (22.7)	5 (21.7)	
** Female–female**	7 (7.6)	5 (7.6)	2 (8.7)	
** Female–male**	44 (47.8)	35 (53.0)	9 (39.1)	
** Missing**	3	3		
**Donor source, *n* (%)**				.789
** MRD**	66 (71.7)	50 (72.5)	16 (69.6)	
** MUD**	26 (28.3)	19 (27.5)	7 (30.4)	
**ABO match, *n* (%)**				.508
** Matched**	50 (54.3)	39 (56.5)	11 (47.8)	
** Minor mismatched**	13 (14.1)	11 (15.9)	2 (8.7)	
** Major mismatched**	17 (18.5)	11 (15.9)	6 (26.1)	
** Major-minor mismatched**	12 (13.0)	8 (11.6)	4 (17.4)	
**Conditioning regimen including Bu, *n* (%)**	30 (32.6)	27 (39.1)	3 (13.0)	.021
**Source of stem cell including BM, *n* (%)**	67 (72.8)	49 (71.0)	18 (78.3)	.499
**Graft composition**				
** Median MNCs counts, ×10^8^/kg (range)**	8.58 (1.41-31.78)	9.56 (4.40-31.78)	7.78 (1.41-15.35)	.005
** Median CD34^+^ cells counts, ×10^6^/kg (range)**	2.64 (0.34-10.44)	2.85 (1.02-10.44)	1.95 (0.34-5.36)	.050

Abbreviations: ATG, antithymocyte globulin; BM, bone marrow; Bu, busulfan; FDC, full donor chimerism; HCT-CI, hematopoietic cell transplantation-specific comorbidity index; HSCT, hematopoietic stem cell transplantation; MC, mixed chimerism; MNCs, mononuclear cells; MRD, HLA-matched related donor; MUD, HLA-matched unrelated donor; RBC, red blood cell; SAA, severe aplastic anemia; vSAA, very severe aplastic anemia.

### Clinical outcomes

**Figure 2.**
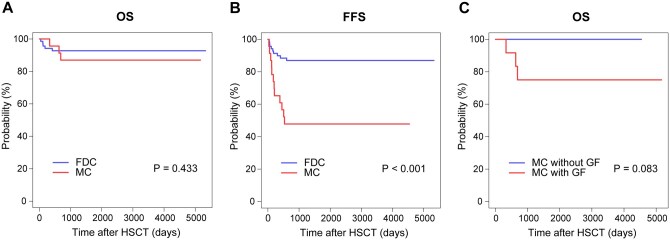
Survival outcomes comparison between the FDC and MC groups and within the MC group. (A) The 5-year OS rates were 92.8% (95% CI, 86.8-99.1) in the FDC group and 87.0% (95% CI, 74.2-100) in the MC group (*P* = .433). (B) The FDC group demonstrated a 5-year FFS of 87.0% (95% CI, 79.4-95.3), significantly higher than 47.8% (95% CI, 31.2-73.3) in the MC group (*P* < .001). (C) In the MC group, patients with GF had a 5-year OS of 75.0% (95% CI, 54.1-100) vs 100% in those without GF, with a non-significant difference (*P* = .083).

The clinical outcomes of patients between the FDC and MC groups were summarized in **Table S1**. All patients in both groups successfully achieved neutrophil engraftment by Day +28 post-HSCT, and the platelet engraftment rates within 100 days were similar (FDC 97.1% vs MC 95.7%, *P* = .958). A total of 18 patients developed GF in this study, with a median onset time of 6.3 months (range, 1.0-20.0 months), and patients with MC had a significantly greater GF incidence (52.2% vs 8.7%, *P* < .001). The incidences of CMV reactivation (56.5% vs 39.1%, *P* = .154) and EBV reactivation (15.9% vs 4.3%, *P* = .164) within 100 days after HSCT were comparable between the FDC and MC groups. In terms of the incidence of aGvHD, no statistically significant differences were identified, although a trend toward higher grade II-IV aGvHD was observed in the FDC group (11.5% vs 0%, *P* = .090). Grade III-IV aGvHD remained infrequent across both groups, with the FDC group showing a 2.9% incidence as opposed to 0% in the MC group (*P* = .412). The 5-year OS rates did not significantly differ, with rates of 92.8% (95% confidence interval [CI], 86.8-99.1) in the FDC group and 87.0% (95% CI, 74.2-100) in the MC group (*P* = .433; Figure 2A). Nevertheless, the FDC group demonstrated a superior 5-year FFS of 87.0% (95% CI, 79.4-95.3), compared with 47.8% (95% CI, 31.2-73.3) in the MC group (*P* < .001; Figure 2B). Notably, in the MC group, patients who developed GF had poorer 5-year OS, although this difference did not reach statistical significance (75.0% [95% CI, 54.1-100] vs 100%, *P* = .083; Figure 2C).

### Dynamic immune reconstitution of cell subsets

**Figure 3.**
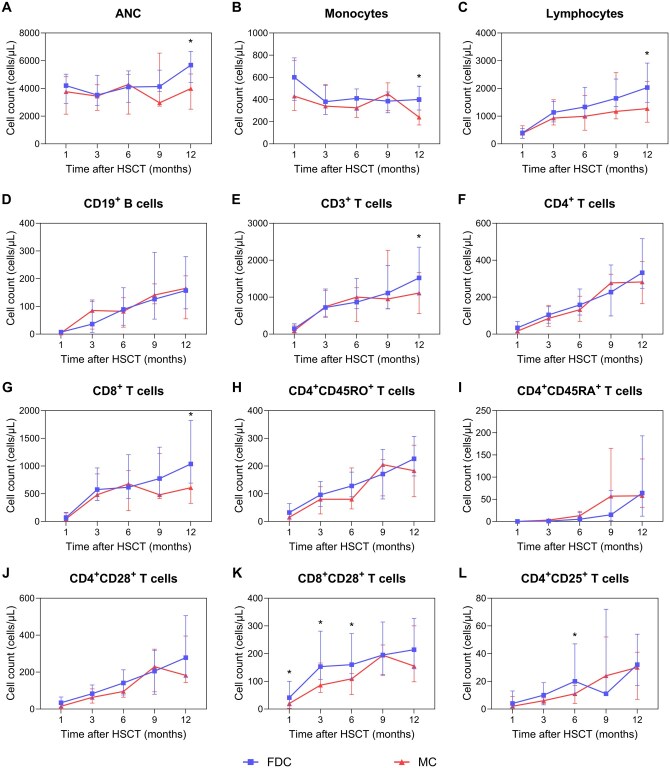
Immune cell subset reconstitution between the FDC and MC groups after HSCT. Error bars indicate the 25th to 75th percentiles. (A-I) At 12 months post-HSCT, the FDC group had significantly higher absolute counts of key myeloid and lymphoid subsets compared to the MC group: ANC: median 5680 (4430-6675) vs 3990 (2505-5040) cells/μL (*P* = .001); monocytes: 400 (305-520) vs 240 (170-380) cells/μL (*P* = .007); lymphocytes: 2030 (1500-2915) vs 1270 (780-2255) cells/μL (*P* = .029); CD3^+^ T cells: 1520 (1058-2354) vs 1111 (554-1663) cells/μL (*P* = .041); CD8^+^ T cells: 1038 (692-1823) vs 609 (327-1073) cells/μL (*P* = .020). No significant differences were observed in absolute counts of CD19^+^ B cells, CD4^+^ T cells, CD4^+^CD45RO^+^ T cells, or CD4^+^CD45RA^+^ T cells. (J-K) The MC group had persistently reduced absolute counts of CD8^+^CD28^+^ T cells at 1, 3, and 6 months post-HSCT: 1 month: 20 (2-42) vs 41 (11-100) cells/μL (*P* = .049); 3 months: 86 (60-166) vs 153 (107-281) cells/μL (*P* = .024); 6 months: 109 (52-128) vs 160 (108-272) cells/μL (*P* = .001). (L) At 6 months post-HSCT, the MC group had fewer absolute CD4^+^CD25^+^ T cells than the FDC group: median 11 (4-17) vs 20 (10-47) cells/μL (*P* = .006).

The immune reconstitution of the cell subsets following transplantation is shown in **Tables S1-S4**. Among these, the key differences between the FDC and MC groups was presented in Table 2. There were no significant differences between the FDC and MC groups in terms of ANC, monocyte, total lymphocyte, CD3^+^ T cell, or CD19^+^ B cell profiles at 1, 3, 6, or 9 months post-HSCT. However, at 12 months post-HSCT, the FDC group presented significantly greater absolute neutrophil (median, 5680 vs 3990 cells/μL, *P* = .001), monocyte (median, 400 vs 240 cells/μL, *P* = .007), lymphocyte (median, 2030 vs 1270 cells/μL, *P* = .029), and CD3^+^ T cell (median, 1520 vs 1111 cells/μL, *P* = .041; Figure 3A-E) counts.

**Table 2.** Key differences in immune cell subset reconstitution between the FDC and MC groups post-transplantation.

Immune subset	FDC group	MC group	*P*-value
	cells/μL, median (25th-75th)	cells/μL, median (25th-75th)	
**1 month**			
** CD8^+^CD28^+^ T cells**	41 (11-100)	20 (2-42)	.049
**3 months**			
** CD8^+^CD28^+^ T cells**	153 (107-281)	86 (60-166)	.024
**6 months**			
** CD8^+^CD28^+^ T cells**	160 (108-272)	109 (52-128)	.001
** CD4^+^CD25^+^ T cells**	20 (10-47)	11 (4-17)	.006
**12 months**			
** ANC**	5680 (4430-6675)	3990 (2505-5040)	.001
** Monocytes**	400 (305-520)	240 (170-380)	.007
** Lymphocytes**	2030 (1500-2915)	1270 (780-2255)	.029
** CD3^+^ T cells**	1520 (1058-2354)	1111 (554-1663)	.041
** CD8^+^ T cells**	1038 (692-1823)	609 (327-1073)	.020

Abbreviations: ANC, absolute neutrophil cell; FDC, full donor chimerism; MC, mixed chimerism.

**Figure 4.**
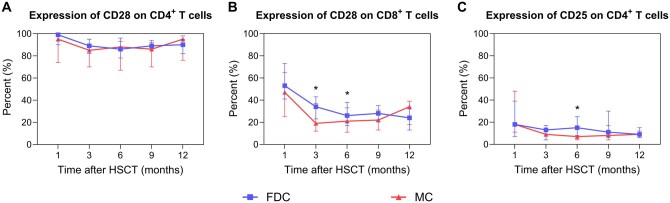
The percent of CD28 and CD25 expression on T cells between the FDC and MC groups after HSCT. Error bars indicate the 25th to 75th percentiles. (A) CD28 expression on CD4^+^ T cells showed no significant differences between the FDC and MC groups. (B) In the MC group, CD28 expression intensity on CD8^+^ T cells was lower at 1, 3, and 6 months (1 month: 0.47 [0.25-0.65] vs 0.53 [0.41-0.73], *P* = .088; 3 months: 0.19 [0.12-0.37] vs 0.34 [0.23-0.43], *P* = .041; 6 months: 0.21 [0.11-0.33] vs 0.26 [0.17-0.38], *P* = .048). (C) At 6 months post-HSCT, the proportion of CD4^+^ T cells expressing CD25 was significantly lower in the MC group compared to the FDC group (0.07 [0.04-0.14] vs 0.15 [0.08-0.25], *P* = .014).

In terms of the T cell subtypes, a predominance of CD8^+^ T cells over CD4^+^ T cells was observed during the 12-month follow-up after transplantation in both analyzed groups. Patients with MC exhibited lower counts of CD8^+^ T cells (median, 609 vs 1038 cells/μL; *P* = .020; Figure 3G). No significant differences were identified between the groups regarding the profiles of CD4^+^ T cells, CD4^+^CD45RO^+^ T cells, or CD4^+^CD45RA^+^ T cells (Figure 3F-I). Notably, immune profiling revealed a marked reduction in CD8^+^CD28^+^ T cell counts in the MC group at 1 month (median, 20 vs 41 cells/μL, *P* = .049), 3 months (median, 86 vs 153 cells/μL, *P* = .024), and 6 months (median, 109 vs 160 cells/μL, *P* = .001) compared with those in the FDC group, a trend not observed in the CD4^+^CD28^+^ T cell subset (Figure 3J-K). Additionally, in the MC group, the expression intensity of CD28 on CD8^+^ T cells remained consistently lower at 1 month (*P* = .088), 3 months (*P* = .041), and 6 months (*P* = .048; Figure 4B). Moreover, at 6 months after transplantation, the number of CD4^+^CD25^+^ T cells was significantly lower in the MC group than in the control group (median, 11 vs 20 cells/μL, *P* = .006; Figure 3L), which was also associated with diminished CD25 expression on CD4^+^ T cells (*P* = .014; Figure 4C).

### Dynamic immune reconstitution of immunoglobulin

**Figure 5.**
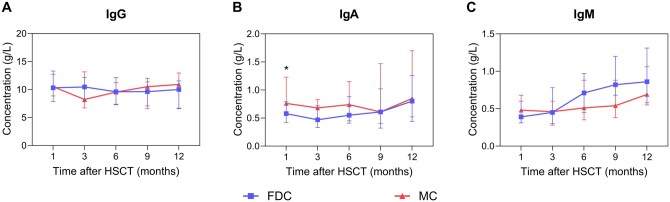
Immunoglobulin reconstitution between the FDC and MC groups after HSCT. Error bars indicate the 25th to 75th percentiles. (A, C) IgG and IgM reconstitution remained comparable between the MC and FDC groups across all evaluated timepoints. (B) Patients with MC exhibited a transient elevation in serum IgA at 1 month post-HSCT compared with those with FDC (median, 0.76 vs 0.58 g/L, *P* = .007), with levels comparable thereafter.

Patients with MC exhibited a transient elevation in serum IgA at 1 month post-HSCT compared with those with FDC (median, 0.76 vs 0.58 g/L, *P* = .007), with levels comparable thereafter (Figure 5B). Importantly, IgG and IgM reconstitution remained comparable between the groups across all evaluated timepoints (Figure 5A and C).

### Impact of immune reconstitution on transplant outcomes

**Figure 6.**
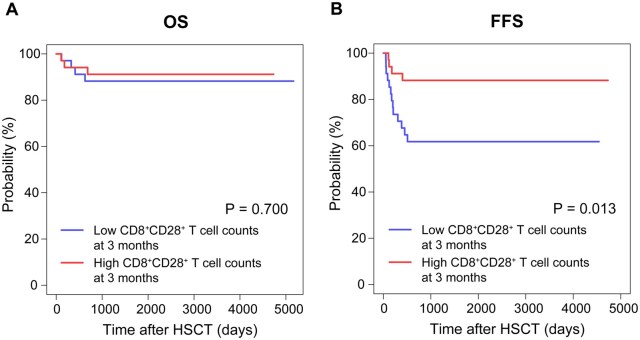
Survival outcomes comparison based on CD8^+^CD28^+^ T cell counts at 3 months. (A) The 5-year OS rate was 91.2% [95% CI, 82.1-100] in the high CD8^+^CD28^+^ T cell count group (≥140 cells/μL) vs 88.2% [95% CI, 78.0-99.8] in the low count group (*P* = .700). (B) The 5-year FFS rate was 88.2% [95% CI, 78.0-99.8] in the high count group and 61.8% [95% CI, 47.4-80.5] in the low count group (*P* = .013).

To investigate the prognostic implications of early immune reconstitution at 1 and 3 months following HSCT, patients were stratified into high- and low-cell count groups based on the median values of various immune cell subsets. The comparative OS and FFS outcomes between these groups are presented in **Table S5**. Notably, although no significant differences were observed in 5-year OS (91.2% [95% CI, 82.1-100] vs 88.2% [95% CI, 78.0-99.8], *P* = .700; Figure 6A), patients with high CD8^+^CD28^+^ T cell counts (≥140 cells/μL) at 3 months presented a significantly improved 5-year FFS compared with those in the low-count group (88.2% [95% CI, 78.0-99.8] vs 61.8% [95% CI, 47.4-80.5], *P* = .013; Figure 6B). Univariate Cox regression analysis revealed that advanced age, Bu-containing conditioning regimens, and high CD8^+^CD28^+^ T cell counts at 3 months were associated with improved FFS (*P* < .10). According to the multivariate analysis, high CD8^+^CD28^+^ T cell counts at 3 months emerged as an independent favorable predictor of FFS (hazard ratio: 0.30 [95% CI, 0.10-0.92], *P* = .035; **Table S6**), highlighting its prognostic significance beyond conventional risk factors.

## Discussion

The occurrence of MC remains a significant yet underrecognized challenge for patients receiving allo-HSCT. Our PSM cohort analysis indicated that MC substantially diminishes FFS, with patients exhibiting MC facing a heightened risk of GF or mortality compared with those with FDC in HLA-matched HSCT settings. Importantly, this study is the first to systematically delineate the immune reconstitution profile in patients with SAA with MC, particularly revealing a selective and profound deficiency in CD8^+^CD28^+^ T cell recovery as the immunological hallmark of this high-risk group.

Patients undergoing HLA-matched transplantation demonstrate a greater incidence of MC than patients undergoing haplo-HSCT do, possibly due to reduced alloreactivity in histocompatible environments and the frequent use of reduced-intensity conditioning regimens that fail to completely eliminate residual host hematopoiesis.^9^^,^^19^ This observation led to our targeted investigation within the MRD/MUD cohort. There are substantial differences in patient outcomes associated with MC. Our analysis revealed no significant difference in OS between the MC and FDC groups (5-year OS: 87.0% vs 92.8%), likely because of effective salvage therapies for complications associated with MC. However, MC was associated with a higher incidence of GF and resulted in a notable 39.2% absolute reduction in 5-year FFS (47.8% vs 87.0%). Consistently, previous studies have also found that MC leads to an increased risk of GF occurrence.^6^^,^^7^ Notably, patients with MC who experienced GF had a nonsignificant trend toward poorer 5-year OS than their GF-free MC counterparts did (75.0% vs 100%), a finding likely limited by the small number of events. This trend is consistent with findings from other centers.^6–8^ For example, Kako et al.^6^ reported the clinical outcomes of patients with SAA from the Japanese Transplant Registry Unified Management Program, indicating that MC with secondary GF was associated with poor 5-year OS (52.1% vs 83.5%, *P* < 0.001), whereas MC without GF demonstrated survival rates comparable to those with FDC.

Previous studies in the field of SAA have recognized age, sex, and graft source as critical factors influencing immune reconstitution following HSCT.^20^^,^^21^ However, the impact of the chimeric status on immune reconstitution in patients with SAA remains insufficiently explored. Although prior studies demonstrated sustained long-term immune functionality in MC patients with nonmalignant disease over a decade post-HSCT, their findings were limited by small sample sizes and a focus on chronic-phase adaptations rather than early dynamic changes.^22^ To specifically investigate the immunological effects of MC during the critical first year post-transplantation, a period of heightened clinical relevance for intervention, we employed PSM to control for confounding factors, thereby establishing balanced cohorts with equivalent baseline immunological risk. Our analysis revealed largely comparable myeloid and lymphoid reconstitution between the MC and FDC cohorts within 9 months post-HSCT. However, by 12 months, patients with FDC exhibited superior recovery of ANC, monocyte, and total lymphocyte counts. This divergence reflects the progressive immune dysregulation observed in patients with MC patients, particularly those progressing to secondary GF, as detailed in the study. Intriguingly, patients with MC demonstrated a transient elevation in serum IgA at 1 month post-HSCT, which subsequently returned comparable thereafter. No significant differences were observed in terms of IgM, IgG, or total B cell counts across any of the timepoints. We propose that the transient elevation in IgA may result from the coexistence of donor and recipient B cells during the early post-transplantation phase in MC patients. Additionally, early intestinal mucosal injury may facilitate the release of secretory IgA into the bloodstream, and the delayed immune reconstitution in MC patients could exacerbate mucosal damage, further promoting IgA leakage.^23^ Further investigation is warranted to explore donor- and recipient-specific IgA clonotypes and secretory IgA in mucosal fluids to elucidate their biological significance in the context of MC. Stikvoort et al.^22^ reported comparable ANC, T cell subset distributions (CD3^+^/CD4^+^/CD8^+^ T cells), B cells, and natural killer (NK) cells between patients with long-term stable MC (*n* = 12) and those with FDC (*n* = 13) with nonmalignant diseases at a median follow-up of 10 years post-HSCT. These findings highlight the importance of closely monitoring cytopenia trends in patients with MC during the first year after transplantation.

A notable defect in adaptive immune reconstitution was identified: patients with MC presented significantly lower absolute counts of CD8^+^CD28^+^ T cells and a lower proportional representation within the CD8^+^ T cell compartment during the first 6 months post-HSCT than FDC controls did, despite having comparable total CD8^+^ T cell numbers between the groups. CD28 is a crucial costimulatory molecule involved in T cell activation. Upon binding to its ligands, CD80 and CD86, which are typically expressed on antigen-presenting cells, CD28 facilitates the delivery of the second signal essential for T lymphocyte activation, thereby enhancing immune responses.^24^ The lack of CD28 expression is associated with terminal differentiation and exhaustion phenotypes, resulting in diminished anti-tumor and anti-infection capabilities.^25^ Furthermore, other studies have identified a correlation between MC and the risk of recurrence in patients with hematological malignancies.^26^ Based on our immune reconstitution results, it can be assumed that lower levels of CD8^+^CD28^+^ T cells lead to attenuation of the graft-versus-leukemia effect. BM damage, induced by the abnormal activation and hyperfunction of T lymphocytes, is recognized as a significant pathogenic mechanism in SAA.^27^ Regrettably, we were unable to ascertain the donor or recipient origin of CD8^+^CD28^+^ T cells via lineage-specific chimerism analysis, which constitutes a significant limitation of the present study. Conducting an assessment of T-cell-specific chimerism would have considerably enhanced the interpretation of our results. Specifically, if the diminished CD8^+^CD28^+^ T cell counts observed in patients with MC were of donor origin, it would suggest a direct impairment in the maturation and expansion of donor T cells, indicative of a defect in donor immune reconstitution following transplantation. Prior research has demonstrated that an increase in host T lymphocytes elevates the risk of GF, and in vitro studies have shown that inhibiting CD28 diminishes the capacity of donor CD8^+^ T cells to mount an immune response against allogeneic antigens.^28^^,^^29^ Consequently, we hypothesize that donor-derived CD8^+^CD28^+^ T cells may play a crucial role in maintaining donor chimerism dominance by targeting residual host autoreactive T cells post-transplantation, which could explain the significantly elevated risk of GF in patients with SAA. It is important to acknowledge, however, that an excessively high proportion of CD8^+^CD28^+^ T cells also poses a risk of inducing GVHD.^30^ The immune reconstitution status of this cell subset after transplantation is of great significance to chimerism formation and tolerance induction of patients. Moreover, early CD8^+^CD28^+^ T cell counts at 3 months not only predicted superior 5-year FFS (88.2% vs 61.8%) but also emerged as an independent protective factor, underscoring their crucial role in maintaining chimeric stability and ensuring long-term transplant success.

In this study, we observed that patients with MC exhibited significantly lower proportions and absolute counts of CD4^+^CD25^+^ T cells at 6 months post-transplantation than FDC controls. This finding suggests that CD4^+^CD25^+^ T cells may play a crucial role in regulating post-transplant chimerism status. Historically, CD4^+^CD25^+^ T cells were the first phenotypic markers used to identify regulatory T cells (Tregs), as early research linked this subset to immune tolerance.^31^ In a similar context, Hanash et al.^32^ demonstrated in a murine transplantation model with major histocompatibility complex-mismatched donors that donor CD4^+^CD25^+^ T cells enhanced engraftment, reduced rejection rates, and prevented GvHD-related manifestations. However, the specificity of CD4^+^CD25^+^ as a Treg marker was later questioned, as activated conventional T cells also upregulate CD25, resulting in heterogeneity within the CD4^+^CD25^+^ population.^33^ This limitation was addressed with the discovery of FOXP3, a forkhead box transcription factor that serves as the master regulator of Treg development and function. Additionally, prior research has confirmed the heterogeneity of CD4^+^CD25^+^ cells, indicating that only the CD25^high^ subset is enriched for true Tregs characterized by FOXP3 expression, whereas the CD25^int^ subset exhibits a low proportion of Tregs.^33^ In clinical practice, however, we assessed CD4^+^CD25^+^ T cells without incorporating FOXP3 co-staining, as this marker combination was not routinely incorporated into our clinical immune monitoring panel for the historical SAA transplant cohort. This methodological limitation constrains the precision of our Treg subset characterization. Despite this constraint, emerging clinical evidence underscores the pivotal role of bona fide Tregs in modulating post-transplant chimerism and GF. Ren et al.^34^ reported a significant reduction in the frequency of Tregs (CD4^+^CD25^+^FOXP3^+^CD127^dim/−^) during secondary GF in a cohort of 16 patients with MC or secondary GF, including five patients with SAA (from 2.66% to 0.93%). Notably, following intervention with donor lymphocyte infusion from alternative donors, MC was successfully converted to FDC in 14 patients (87.5%), which was accompanied by a gradual normalization of neutrophil and platelet counts. Concurrently, Treg frequencies increased from 0.92% during SGF to 3.61%, suggesting that exogenous lymphocyte infusion may reverse mixed chimerism by restoring Treg homeostasis.

When analyzing differences in T cell reconstitution, it is important to consider the potential association with Bu-containing conditioning regimens. Previous research, including our own, has identified Bu as a protective factor against MC, and the present study further demonstrates that the MC group exhibited both reduced Bu exposure and delayed reconstitution of specific T cell subsets.^9^^,^^35^^,^^36^ We propose that Bu may indirectly influence MC development by modulating immune reconstitution; however, this association lacks direct empirical validation. And the possibility of Bu having a direct effect on immune reconstitution cannot be excluded, necessitating further research to elucidate this relationship.

Several limitations of this study must be acknowledged. Firstly, the functional significance of the observed quantitative deficiencies in CD8^+^CD28^+^ and CD4^+^CD25^+^ T cell subsets remains uncertain due to the lack of functional assays, such as assessments of T cell proliferation and cytotoxic activity. Additionally, our chimerism analysis utilized unfractionated samples without lineage-specific evaluation, such as T cell chimerism, which precludes the determination of the donor or recipient origin of these T cells. Secondly, the dynamics of NK cells, including cytotoxicity markers and subset distributions, were not systematically monitored, resulting in a gap in the assessment of their role in MC-related risk, especially given their involvement in promoting engraftment and immune tolerance.^37^^,^^38^ It is imperative to incorporate comprehensive NK cell monitoring in future prospective studies. Thirdly, the present study was limited to the quantification of CD4^+^CD25^+^ T cells alone, without simultaneous assessment of FOXP3 expression, which was critical for defining bona fide Tregs. Future research should incorporate multi-marker phenotyping (combining CD4, CD25, and FOXP3) to precisely identify Treg cells and validate the role of Treg dyshomeostasis in the maintenance of MC. Finally, the modest sample size of the MC cohort and the heterogeneous timing of chimerism conversion may obscure subgroup-specific trends, particularly in correlating dynamic immune reconstitution trajectories with late-phase events such as secondary GF or infection-related mortality.

In conclusion, this study provides the first comprehensive elucidation of posttransplant immune reconstitution dynamics in patients with SAA with MC, demonstrating that the suppressed status of CD8^+^CD28^+^ T cells and CD4^+^CD25^+^ T cell subsets in the MC cohort may critically contribute to GF. Future research should prioritize the optimization of conditioning regimens and the mitigation of risk factors to enhance the recovery of donor-derived immune subsets, thereby reducing MC prevalence and GF rates while improving long-term outcomes for patients with SAA.

## Supplementary Material

szag009_Supplementary_Data

## Data Availability

The datasets generated during and/or analysed during the current study are available from the corresponding author on reasonable request.
