# Supplementary material for: Dynamic immune reconstitution and clinical outcomes in different chimerism statuses of HLA-matched transplantation for severe aplastic anemia
Source: Stem Cells Transl Med. 2026 Mar 27;15(4):szag009. doi: 10.1093/stcltm/szag009 (PMC13026414; doi:10.1093/stcltm/szag009)
Supplement: szag009_Supplementary_Data [file szag009_supplementary_data.docx]

**Supplementary Information**

**Dynamic immune reconstitution and clinical outcomes in different chimerism statuses of HLA-matched transplantation for severe aplastic anemia**

Ming-Hao Lin1#, Zheng-Li Xu1#, Ying-Jun Chang1, Hui-Dong Guo1, Lan-Ping Xu1, Yu Wang1, Xiao-Hui Zhang1, Yi-Fei Cheng1, Yuan-Yuan Zhang1, Xiao-Dong Mo1, Yu-Qian Sun1, Ting-Ting Han1, Jing-Zhi Wang1, Yao Chen1, Yu-Hong Chen1, Huan Chen1, Wei Han1, Xiao-Jun Huang1,2*

# Equal contribution.

**Author Affiliations**

1 Peking University People’s Hospital, Peking University Institute of Hematology, National Clinical Research Center for Hematologic Disease, Beijing Key Laboratory of Cell and Gene Therapy for Hematologic Malignancies, Peking University, Beijing, China.

2 Peking-Tsinghua Center for Life Sciences, Academy for Advanced Interdisciplinary Studies, Peking University, Beijing, China.

* Corresponding author

Prof. Xiao-Jun Huang; Peking University Institute of Hematology, Peking University People’s Hospital, No. 11 Xizhimen South Street, Xicheng District, Beijing 100044, P.R.China; Tel: 8610-8832-6006; E-mail: huangxiaojun@bjmu.edu.cn.

**Supplementary Appendix**

**[Table S1](#_Toc19811)**[. The values of immune cell subset counts post-transplantation between the FDC and MC groups. 3](#_Toc19811)

**[Table S2](#_Toc1153)**[. Comparison of immune cell subset counts post-transplantation between patients receiving Bu-containing and non-Bu conditioning regimens. 6](#_Toc1153)

**[Table S3](#_Toc23477)**[. Correlation between graft cell doses and immune cell subset counts at different time points post-transplantation. 9](#_Toc23477)

**[Table S4](#_Toc18917)**[. The propotions of immune cell subset post-transplantation between the FDC and MC groups. 12](#_Toc18917)

**[Table S5](#_Toc10141)**[. The comparative OS and FFS outcomes stratified by immune cell subsets post-transplantation. 14](#_Toc10141)

**[Table S6](#_Toc19083)**[. Multivariate analysis of risk factors for FFS. 17](#_Toc19083)

**Table S1**. The values of immune cell subset counts post-transplantation between the FDC and MC groups.

| **Immune cell subset** | **FDC group** | **MC group** | **P value** |
| --- | --- | --- | --- |
|  | **cells/μL, median (25^th^-75^th^)** | **cells/μL, median (25^th^-75^th^)** |  |
| 1 month |  |  |  |
| ANC | 4200 (2925–5030) | 3765 (2140–4843) | 0.353 |
| Monocytes | 600 (393–775) | 430 (300–750) | 0.421 |
| Lymphocytes | 395 (200–553) | 380 (200–650) | 0.542 |
| CD19^+^ B cells | 7 (3–14) | 4 (3–10) | 0.168 |
| CD3^+^ T cells | 148 (36–279) | 97 (11–243) | 0.317 |
| CD4^+^ T cells | 34 (9–68) | 16 (1–41) | 0.083 |
| CD8^+^ T cells | 71 (20–164) | 47 (7–153) | 0.505 |
| CD4^+^CD45RO^+^ T cells | 32 (8–64) | 15 (1–35) | 0.053 |
| CD4^+^CD45RA^+^ T cells | 0 (0–1) | 0 (0–1) | 0.256 |
| CD4^+^CD28^+^ T cells | 34 (8–65) | 15 (1–41) | 0.058 |
| CD8^+^CD28^+^ T cells | 41 (11–100) | 20 (2–42) | 0.049 |
| CD4^+^CD25^+^ T cells | 4 (1–13) | 2 (1–9) | 0.266 |
| 3 months |  |  |  |
| ANC | 3520 (2758–4950) | 3440 (2415–4265) | 0.416 |
| Monocytes | 380 (265–525) | 340 (265–535) | 0.834 |
| Lymphocytes | 1135 (793–1533) | 930 (680–1590) | 0.648 |
| CD19^+^ B cells | 36 (6–123) | 85 (18–118) | 0.337 |
| CD3^+^ T cells | 722 (456–1221) | 743 (483–1186) | 0.840 |
| CD4^+^ T cells | 104 (59–148) | 85 (42–155) | 0.536 |
| CD8^+^ T cells | 576 (378–965) | 483 (379–857) | 0.710 |
| CD4^+^CD45RO^+^ T cells | 96 (54–144) | 80 (27–125) | 0.234 |
| CD4^+^CD45RA^+^ T cells | 1 (0–4) | 3 (1–4) | 0.332 |
| CD4^+^CD28^+^ T cells | 83 (53–130) | 63 (32–109) | 0.246 |
| CD8^+^CD28^+^ T cells | 153 (107–281) | 86 (60–166) | 0.024 |
| CD4^+^CD25^+^ T cells | 10 (3–19) | 6 (4–8) | 0.159 |
| 6 months |  |  |  |
| ANC | 4100 (3005–5260) | 4290 (2148–5015) | 0.235 |
| Monocytes | 410 (300–495) | 325 (238–413) | 0.088 |
| Lymphocytes | 1330 (965–2040) | 995 (493–1740) | 0.099 |
| CD19^+^ B cells | 89 (32–167) | 82 (25–131) | 0.661 |
| CD3^+^ T cells | 866 (687–1508) | 1001 (336–1253) | 0.264 |
| CD4^+^ T cells | 158 (103–244) | 132 (69–205) | 0.229 |
| CD8^+^ T cells | 618 (417–1204) | 678 (194–915) | 0.329 |
| CD4^+^CD45RO^+^ T cells | 128 (89–178) | 80 (45–193) | 0.073 |
| CD4^+^CD45RA^+^ T cells | 5 (2–20) | 13 (4–23) | 0.239 |
| CD4^+^CD28^+^ T cells | 141 (76–212) | 95 (64–130) | 0.084 |
| CD8^+^CD28^+^ T cells | 160 (108–272) | 109 (52–128) | 0.001 |
| CD4^+^CD25^+^ T cells | 20 (10–47) | 11 (4–17) | 0.006 |
| 9 months |  |  |  |
| ANC | 4135 (3778–5325) | 2960 (2720–6550) | 0.450 |
| Monocytes | 385 (280–468) | 450 (300–550) | 0.449 |
| Lymphocytes | 1640 (1340–2338) | 1170 (900–2570) | 0.395 |
| CD19^+^ B cells | 126 (54–295) | 141 (109–181) | 0.777 |
| CD3^+^ T cells | 1112 (680–1857) | 951 (688–2269) | 0.962 |
| CD4^+^ T cells | 227 (98–374) | 277 (229–324) | 0.369 |
| CD8^+^ T cells | 774 (463–1341) | 481 (412–1226) | 0.637 |
| CD4^+^CD45RO^+^ T cells | 171 (81–260) | 205 (92–223) | 0.683 |
| CD4^+^CD45RA^+^ T cells | 15 (2–70) | 57 (14–165) | 0.103 |
| CD4^+^CD28^+^ T cells | 205 (80–318) | 229 (93–324) | 0.777 |
| CD8^+^CD28^+^ T cells | 195 (124–314) | 194 (121–231) | 0.395 |
| CD4^+^CD25^+^ T cells | 22 (10–72) | 24 (11–52) | 0.962 |
| 12 months |  |  |  |
| ANC | 5680 (4430–6675) | 3990 (2505–5040) | 0.001 |
| Monocytes | 400 (305–520) | 240 (170–380) | 0.007 |
| Lymphocytes | 2030 (1500–2915) | 1270 (780–2255) | 0.029 |
| CD19^+^ B cells | 157 (91–279) | 165 (55–210) | 0.503 |
| CD3^+^ T cells | 1520 (1058–2354) | 1111 (554–1663) | 0.041 |
| CD4^+^ T cells | 332 (248–516) | 282 (164–392) | 0.242 |
| CD8^+^ T cells | 1038 (692–1823) | 609 (327–1073) | 0.020 |
| CD4^+^CD45RO^+^ T cells | 226 (164–307) | 183 (89–275) | 0.104 |
| CD4^+^CD45RA^+^ T cells | 64 (12–193) | 58 (32–141) | 0.670 |
| CD4^+^CD28^+^ T cells | 278 (172–505) | 182 (143–395) | 0.354 |
| CD8^+^CD28^+^ T cells | 214 (145–327) | 155 (98–300) | 0.183 |
| CD4^+^CD25^+^ T cells | 32 (17–54) | 30 (7–41) | 0.444 |

*Abbreviations: ANC, absolute nuclear cell; FDC, full donor chimerism; MC, mixed donor chimerism.*

**Table S2**. Comparison of immune cell subset counts post-transplantation between patients receiving Bu-containing and non-Bu conditioning regimens.

| **Immune cell subset** | **No Bu** | **Bu** | **P value** |
| --- | --- | --- | --- |
|  | **cells/μL, median (25^th^-75^th^)** | **cells/μL, median (25^th^-75^th^)** |  |
| 1 month |  |  |  |
| ANC | 3275 (2503–4785) | 4100 (3310–4800) | 0.067 |
| Monocytes | 430 (200–500) | 600 (400–690) | 0.474 |
| Lymphocytes | 300 (200–500) | 400 (130–500) | 0.676 |
| CD19^+^ B cells | 6 (3–11) | 12 (3–20) | 0.428 |
| CD3^+^ T cells | 104 (36–195) | 97 (7–330) | 0.862 |
| CD4^+^ T cells | 24 (4–53) | 12 (1–59) | 0.609 |
| CD8^+^ T cells | 58 (18–116) | 51 (4–223) | 0.888 |
| CD4^+^CD45RO^+^ T cells | 22 (4–45) | 11 (1–53) | 0.597 |
| CD4^+^CD45RA^+^ T cells | 0 (0–1) | 0 (0–1) | 0.543 |
| CD4^+^CD28^+^ T cells | 24 (4–49) | 10 (1–60) | 0.511 |
| CD8^+^CD28^+^ T cells | 33 (10–51) | 29 (2–102) | 0.582 |
| CD4^+^CD25^+^ T cells | 3 (1–9) | 4 (0–13) | 0.633 |
| 3 months |  |  |  |
| ANC | 3240 (2398–4300) | 4115 (3083–4750) | 0.094 |
| Monocytes | 310 (218–500) | 485 (320–633) | 0.046 |
| Lymphocytes | 970 (680–1553) | 1015 (785–1255) | 0.086 |
| CD19^+^ B cells | 35 (14–115) | 65 (3–139) | 0.088 |
| CD3^+^ T cells | 675 (483–1253) | 771 (419–1156) | 1.000 |
| CD4^+^ T cells | 86 (48–143) | 94 (72–160) | 0.287 |
| CD8^+^ T cells | 530 (380–1079) | 576 (364–890) | 0.942 |
| CD4^+^CD45RO^+^ T cells | 83 (34–139) | 92 (67–146) | 0.364 |
| CD4^+^CD45RA^+^ T cells | 1 (0–4) | 2 (0–3) | 0.859 |
| CD4^+^CD28^+^ T cells | 72 (29–111) | 80 (56–136) | 0.346 |
| CD8^+^CD28^+^ T cells | 137 (81–210) | 144 (84–226) | 0.655 |
| CD4^+^CD25^+^ T cells | 7 (3–18) | 8 (2–12) | 0.667 |
| 6 months |  |  |  |
| ANC | 4360 (2920–5070) | 4245 (3045–5015) | 0.854 |
| Monocytes | 360 (240–450) | 405 (300–513) | 0.288 |
| Lymphocytes | 1090 (860–1520) | 1320 (995–1853) | 0.211 |
| CD19^+^ B cells | 83 (41–134) | 105 (31–185) | 0.467 |
| CD3^+^ T cells | 735 (451–1168) | 1083 (762–1475) | 0.067 |
| CD4^+^ T cells | 132 (78–212) | 185 (133–258) | 0.063 |
| CD8^+^ T cells | 486 (338–857) | 754 (564–1063) | 0.067 |
| CD4^+^CD45RO^+^ T cells | 101 (61–143) | 158 (122–218) | 0.008 |
| CD4^+^CD45RA^+^ T cells | 8 (3–48) | 8 (3–20) | 0.502 |
| CD4^+^CD28^+^ T cells | 104 (61–182) | 154 (110–209) | 0.076 |
| CD8^+^CD28^+^ T cells | 109 (52–146) | 217 (121–274) | <0.001 |
| CD4^+^CD25^+^ T cells | 13 (8–27) | 17 (10–26) | 0.436 |
| 9 months |  |  |  |
| ANC | 3965 (2810–4610) | 4875 (4468–5743) | 0.049 |
| Monocytes | 385 (288–443) | 430 (285–478) | 0.541 |
| Lymphocytes | 1255 (808–2098) | 1640 (1475–2418) | 0.121 |
| CD19^+^ B cells | 100 (10–152) | 242 (177–308) | 0.006 |
| CD3^+^ T cells | 975 (679–1451) | 1091 (891–1780) | 0.533 |
| CD4^+^ T cells | 244 (114–325) | 268 (135–383) | 0.746 |
| CD8^+^ T cells | 673 (464–1118) | 834 (472–1380) | 0.566 |
| CD4^+^CD45RO^+^ T cells | 182 (98–229) | 187 (129–297) | 0.683 |
| CD4^+^CD45RA^+^ T cells | 21 (10–102) | 4 (3–43) | 0.367 |
| CD4^+^CD28^+^ T cells | 195 (92–301) | 230 (120–332) | 0.746 |
| CD8^+^CD28^+^ T cells | 187 (120–247) | 199 (166–255) | 0.709 |
| CD4^+^CD25^+^ T cells | 19 (10–52) | 32 (12–40) | 0.980 |
| 12 months |  |  |  |
| ANC | 5070 (3400–6430) | 4845 (3820–5538) | 0.649 |
| Monocytes | 340 (235–445) | 375 (308–493) | 0.216 |
| Lymphocytes | 1840 (1270–2805) | 1950 (1548–2260) | 0.757 |
| CD19^+^ B cells | 138 (100–263) | 231 (132–337) | 0.134 |
| CD3^+^ T cells | 1445 (956–2232) | 1276 (1013–1726) | 0.779 |
| CD4^+^ T cells | 351 (206–651) | 314 (238–422) | 0.469 |
| CD8^+^ T cells | 842 (531–1466) | 873 (503–1386) | 0.926 |
| CD4^+^CD45RO^+^ T cells | 196 (155–343) | 220 (152–306) | 0.353 |
| CD4^+^CD45RA^+^ T cells | 96 (40–213) | 56 (9–140) | 0.924 |
| CD4^+^CD28^+^ T cells | 327 (164–565) | 264 (175–414) | 0.523 |
| CD8^+^CD28^+^ T cells | 184 (131–359) | 186 (149–249) | 0.899 |
| CD4^+^CD25^+^ T cells | 32 (13–62) | 26 (20–33) | 0.353 |

*Abbreviations: ANC, absolute nuclear cell; Bu, busulfan; FDC, full donor chimerism; MC, mixed donor chimerism.*

**Table S3**. Correlation between graft cell doses and immune cell subset counts at different time points post-transplantation.

| **Immune cell subset** | **MNCs dose (×10^8^/kg)** | | **CD34^+^ cells dose (×10^6^/kg)** | |
| --- | --- | --- | --- | --- |
|  | **Spearman’s r** | **P value** | **Spearman’s r** | **P value** |
| 1 month |  |  |  |  |
| ANC | 0.136 | 0.247 | 0.210 | 0.077 |
| Monocytes | 0.011 | 0.924 | 0.177 | 0.136 |
| Lymphocytes | -0.179 | 0.131 | 0.006 | 0.960 |
| CD19^+^ B cells | 0.110 | 0.350 | -0.034 | 0.778 |
| CD3^+^ T cells | -0.268 | 0.021 | 0.038 | 0.752 |
| CD4^+^ T cells | -0.280 | 0.016 | 0.094 | 0.431 |
| CD8^+^ T cells | -0.278 | 0.016 | 0.031 | 0.795 |
| CD4^+^CD45RO^+^ T cells | -0.300 | 0.010 | 0.098 | 0.481 |
| CD4^+^CD45RA^+^ T cells | -0.193 | 0.104 | 0.043 | 0.722 |
| CD4^+^CD28^+^ T cells | -0.294 | 0.011 | 0.098 | 0.413 |
| CD8^+^CD28^+^ T cells | -0.317 | 0.006 | 0.016 | 0.893 |
| CD4^+^CD25^+^ T cells | -0.149 | 0.204 | 0.153 | 0.201 |
| 3 months |  |  |  |  |
| ANC | 0.312 | 0.010 | 0.043 | 0.732 |
| Monocytes | 0.009 | 0.945 | 0.051 | 0.689 |
| Lymphocytes | 0.155 | 0.212 | -0.050 | 0.692 |
| CD19^+^ B cells | 0.022 | 0.857 | -0.125 | 0.323 |
| CD3^+^ T cells | -0.067 | 0.590 | -0.080 | 0.526 |
| CD4^+^ T cells | 0.012 | 0.924 | -0.068 | 0.591 |
| CD8^+^ T cells | -0.059 | 0.634 | -0.041 | 0.745 |
| CD4^+^CD45RO^+^ T cells | 0.055 | 0.664 | -0.032 | 0.806 |
| CD4^+^CD45RA^+^ T cells | -0.156 | 0.211 | -0.149 | 0.240 |
| CD4^+^CD28^+^ T cells | 0.022 | 0.861 | -0.034 | 0.786 |
| CD8^+^CD28^+^ T cells | -0.031 | 0.806 | -0.080 | 0.526 |
| CD4^+^CD25^+^ T cells | -0.349 | 0.004 | 0.008 | 0.951 |
| 6 months |  |  |  |  |
| ANC | 0.100 | 0.432 | 0.111 | 0.393 |
| Monocytes | 0.058 | 0.647 | -0.045 | 0.726 |
| Lymphocytes | 0.026 | 0.837 | -0.081 | 0.531 |
| CD19^+^ B cells | 0.142 | 0.262 | 0.111 | 0.392 |
| CD3^+^ T cells | 0.033 | 0.793 | -0.075 | 0.561 |
| CD4^+^ T cells | -0.015 | 0.905 | -0.076 | 0.558 |
| CD8^+^ T cells | 0.052 | 0.684 | -0.031 | 0.811 |
| CD4^+^CD45RO^+^ T cells | 0.030 | 0.819 | -0.112 | 0.402 |
| CD4^+^CD45RA^+^ T cells | -0.001 | 0.995 | -0.014 | 0.915 |
| CD4^+^CD28^+^ T cells | 0.020 | 0.878 | 0.020 | 0.879 |
| CD8^+^CD28^+^ T cells | 0.124 | 0.330 | 0.098 | 0.449 |
| CD4^+^CD25^+^ T cells | 0.148 | 0.249 | 0.077 | 0.553 |
| 9 months |  |  |  |  |
| ANC | 0.419 | 0.030 | 0.223 | 0.274 |
| Monocytes | 0.222 | 0.267 | 0.200 | 0.328 |
| Lymphocytes | 0.380 | 0.050 | -0.039 | 0.849 |
| CD19^+^ B cells | 0.576 | 0.002 | 0.154 | 0.452 |
| CD3^+^ T cells | 0.190 | 0.344 | -0.024 | 0.906 |
| CD4^+^ T cells | 0.154 | 0.444 | -0.170 | 0.407 |
| CD8^+^ T cells | 0.190 | 0.343 | 0.059 | 0.774 |
| CD4^+^CD45RO^+^ T cells | 0.312 | 0.128 | 0.055 | 0.799 |
| CD4^+^CD45RA^+^ T cells | -0.020 | 0.926 | -0.439 | 0.032 |
| CD4^+^CD28^+^ T cells | 0.155 | 0.439 | -0.186 | 0.362 |
| CD8^+^CD28^+^ T cells | 0.042 | 0.834 | -0.035 | 0.864 |
| CD4^+^CD25^+^ T cells | 0.077 | 0.730 | -0.138 | 0.500 |
| 12 months |  |  |  |  |
| ANC | 0.235 | 0.082 | 0.120 | 0.391 |
| Monocytes | 0.171 | 0.207 | 0.174 | 0.214 |
| Lymphocytes | 0.065 | 0.635 | 0.051 | 0.719 |
| CD19^+^ B cells | 0.159 | 0.240 | 0.069 | 0.623 |
| CD3^+^ T cells | -0.166 | 0.222 | -0.049 | 0.730 |
| CD4^+^ T cells | -0.191 | 0.158 | 0.097 | 0.490 |
| CD8^+^ T cells | -0.151 | 0.267 | -0.055 | 0.695 |
| CD4^+^CD45RO^+^ T cells | -0.152 | 0.269 | 0.081 | 0.570 |
| CD4^+^CD45RA^+^ T cells | -0.098 | 0.478 | 0.041 | 0.775 |
| CD4^+^CD28^+^ T cells | -0.196 | 0.148 | 0.100 | 0.477 |
| CD8^+^CD28^+^ T cells | -0.347 | 0.007 | 0.030 | 0.828 |
| CD4^+^CD25^+^ T cells | -0.217 | 0.109 | 0.043 | 0.760 |

*Abbreviations: ANC, absolute nuclear cell; Bu, busulfan; FDC, full donor chimerism; MC, mixed donor chimerism; MNCs,mononuclear cells.*

**Table S4**. The propotions of immune cell subset post-transplantation between the FDC and MC groups.

| **Immune cell subset** | **FDC group** | **MC group** | **P value** |
| --- | --- | --- | --- |
|  | **median (25^th^-75^th^)** | **median (25^th^-75^th^)** |  |
| 1 month |  |  |  |
| CD4/CD8 | 0.37 (0.21-0.64) | 0.24 (0.15-0.49) | 0.061 |
| CD4 expression on CD3^+^ T cells | 0.21 (0.13-0.32) | 0.14 (0.0-0.22) | 0.060 |
| CD8 expression on CD3^+^ T cells | 0.57 (0.44-0.67) | 0.59 (0.47-0.73) | 0.596 |
| CD28 expression on CD4^+^ T cells | 0.99 (0.90-1.00) | 0.95 (0.74-1.00) | 0.064 |
| CD28 expression on CD8^+^ T cells | 0.53 (0.41-0.73) | 0.47 (0.25-0.65) | 0.088 |
| CD25 expression on CD4^+^ T cells | 0.18 (0.07-0.39) | 0.18 (0.11-0.48) | 0.427 |
| 3 months |  |  |  |
| CD4/CD8 | 0.18 (0.11-0.24) | 0.20 (0.08-0.23) | 0.588 |
| CD4 expression on CD3^+^ T cells | 0.14 (0.10-0.18) | 0.13 (0.07-0.16) | 0.285 |
| CD8 expression on CD3^+^ T cells | 0.80 (0.73-0.85) | 0.78 (0.63-0.87) | 0.804 |
| CD28 expression on CD4^+^ T cells | 0.89 (0.82-0.95) | 0.85 (0.70-0.95) | 0.265 |
| CD28 expression on CD8^+^ T cells | 0.34 (0.23-0.43) | 0.19 (0.12-0.37) | 0.041 |
| CD25 expression on CD4^+^ T cells | 0.13 (0.04-0.17) | 0.09 (0.04-0.14) | 0.515 |
| 6 months |  |  |  |
| CD4/CD8 | 0.21 (0.15-0.40) | 0.28 (0.17-0.40) | 0.524 |
| CD4 expression on CD3^+^ T cells | 0.16 (0.12-0.26) | 0.20 (0.14-0.25) | 0.534 |
| CD8 expression on CD3^+^ T cells | 0.73 (0.64-0.83) | 0.72 (0.62-0.81) | 0.591 |
| CD28 expression on CD4^+^ T cells | 0.86 (0.78-0.96) | 0.88 (0.67-0.93) | 0.543 |
| CD28 expression on CD8^+^ T cells | 0.26 (0.17-0.38) | 0.21 (0.11-0.33) | 0.048 |
| CD25 expression on CD4^+^ T cells | 0.15 (0.08-0.25) | 0.07 (0.04-0.14) | 0.014 |
| 9 months |  |  |  |
| CD4/CD8 | 0.24 (0.17-0.40) | 0.59 (0.20-0.67) | 0.186 |
| CD4 expression on CD3^+^ T cells | 0.18 (0.14-0.26) | 0.32 (0.15-0.37) | 0.202 |
| CD8 expression on CD3^+^ T cells | 0.75 (0.64-0.82) | 0.57 (0.51-0.81) | 0.119 |
| CD28 expression on CD4^+^ T cells | 0.89 (0.87-0.94) | 0.86 (0.70-0.92) | 0.395 |
| CD28 expression on CD8^+^ T cells | 0.28 (0.21-0.35) | 0.22 (0.13-0.31) | 0.257 |
| CD25 expression on CD4^+^ T cells | 0.11 (0.07-0.30) | 0.08 (0.04-0.17) | 0.143 |
| 12 months |  |  |  |
| CD4/CD8 | 0.29 (0.20-0.62) | 0.47 (0.29-0.66) | 0.089 |
| CD4 expression on CD3^+^ T cells | 0.20 (0.16-0.32) | 0.30 (0.19-0.36) | 0.136 |
| CD8 expression on CD3^+^ T cells | 0.72 (0.58-0.79) | 0.61 (0.55-0.65) | 0.075 |
| CD28 expression on CD4^+^ T cells | 0.90 (0.82-0.95) | 0.95 (0.76-0.98) | 0.371 |
| CD28 expression on CD8^+^ T cells | 0.24 (0.13-0.32) | 0.34 (0.18-0.39) | 0.158 |
| CD25 expression on CD4^+^ T cells | 0.09 (0.06-0.15) | 0.09 (0.06-0.12) | 0.828 |

*Abbreviations: FDC, full donor chimerism; MC, mixed donor chimerism.*

**Table S5**. The comparative OS and FFS outcomes stratified by immune cell subsets post-transplantation.

| **Immune subset** | **Group** | **5-year OS** | | **5-year FFS** | |
| --- | --- | --- | --- | --- | --- |
|  |  | **Incidence (95% CI)** | **P value** | **Incidence (95% CI)** | **P value** |
| **1 month** | | | | | |
| CD19^+^ B cells | High (≥ 7 cells/uL) | 83.8% (72.7–96.5%) | 0.041 | 70.3% (57.0–86.7%) | 0.222 |
|  | Low (< 7 cells/uL) | 97.4% (92.4–100%) |  | 81.6% (70.1–94.9%) |  |
| CD3^+^ T cells | High (≥ 103 cells/uL) | 86.5% (76.1–98.%) | 0.215 | 78.4% (66.2–92.8%) | 0.716 |
|  | Low (< 103 cells/uL) | 94.7% (87.9–100%) |  | 73.7% (60.9–89.1%) |  |
| CD4^+^ T cells | High (≥ 23 cells/uL) | 89.2% (79.7–99.8%) | 0.667 | 81.1% (69.4–94.7%) | 0.325 |
|  | Low (< 23 cells/uL) | 92.1% (83.9–100%) |  | 71.1% (58.0–87.0%) |  |
| CD8^+^ T cells | High (≥ 56 cells/uL) | 86.5% (76.1–98.2%) | 0.215 | 78.4% (66.2–92.8%) | 0.716 |
|  | Low (< 56 cells/uL) | 94.7% (87.9–100%) |  | 73.7% (60.9–89.1%) |  |
| CD4^+^CD45RO^+^ T cells | High (≥ 20 cells/uL) | 89.2% (79.7–99.8%) | 0.722 | 78.4% (66.2–92.8%) | 0.544 |
|  | Low (< 20 cells/uL) | 91.7% (83.1–100%) |  | 72.2% (59.0–88.4%) |  |
| CD4^+^CD45RA^+^ T cells | High (≥ 0.2 cells/uL) | 84.6% (74.0–96.7%) | 0.071 | 71.8% (59.0–87.4%) | 0.459 |
|  | Low (< 0.2 cells/uL) | 97.1% (91.5–100%) |  | 79.4% (66.9–94.2%) |  |
| CD4^+^CD28^+^ T cells | High (≥ 22 cells/uL) | 89.2% (79.7–99.8%) | 0.667 | 78.4% (66.2–92.8%) | 0.636 |
|  | Low (< 22 cells/uL) | 92.1% (83.9–100%) |  | 73.7% (60.9–89.1%) |  |
| CD8^+^CD28^+^ T cells | High (≥ 33 cells/uL) | 86.5% (76.1–98.2%) | 0.215 | 73.0% (60.0–88.8%) | 0.525 |
|  | Low (< 33 cells/uL) | 94.7% (87.9–100%) |  | 78.9% (67.0–93.0%) |  |
| CD4^+^CD25^+^ T cells | High (≥ 3 cells/uL) | 89.2% (79.7–99.8%) | 0.667 | 81.1% (69.4–94.7%) | 0.300 |
|  | Low (< 3 cells/uL) | 92.1% (83.9–100%) |  | 71.1% (58.0–87.0%) |  |
| **3 months** | | | | | |
| CD19^+^ B cells | High (≥ 37 cells/uL) | 82.4% (70.5–96.2%) | 0.049 | 70.6% (56.8–87.7%) | 0.380 |
|  | Low (< 37 cells/uL) | 97.1% (91.5–100%) |  | 79.4% (66.9–94.2%) |  |
| CD3^+^ T cells | High (≥ 706 cells/uL) | 91.2% (82.1–100%) | 0.701 | 67.6% (53.6–85.3%) | 0.135 |
|  | Low (< 706 cells/uL) | 88.2% (78.0–99.8%) |  | 82.4% (70.5–96.2%) |  |
| CD4^+^ T cells | High (≥ 92 cells/uL) | 88.2% (78.0–99.8%) | 0.658 | 70.6% (56.8–87.7%) | 0.351 |
|  | Low (< 92 cells/uL) | 91.2% (82.1–100%) |  | 79.4% (66.9–94.2%) |  |
| CD8^+^ T cells | High (≥ 561 cells/uL) | 91.2% (82.1–100%) | 0.701 | 67.6% (53.6–85.3%) | 0.135 |
|  | Low (< 561 cells/uL) | 88.2% (78.0–99.8%) |  | 82.4% (70.5–96.2%) |  |
| CD4^+^CD45RO^+^ T cells | High (≥ 88 cells/uL) | 87.9% (77.4–99.8%) | 0.656 | 69.7% (55.7–87.3%) | 0.223 |
|  | Low (< 88 cells/uL) | 90.9% (81.6–100%) |  | 81.8% (69.7–96.1%) |  |
| CD4^+^CD45RA^+^ T cells | High (≥ 1.1 cells/uL) | 88.2% (78.0–99.8%) | 0.687 | 76.5% (63.5–92.1%) | 0.830 |
|  | Low (< 1.1 cells/uL) | 90.9% (81.6–100%) |  | 72.7% (59.0–89.6%) |  |
| CD4^+^CD28^+^ T cells | High (≥ 75 cells/uL) | 88.2% (78.0–99.8%) | 0.658 | 70.6% (56.8–87.7%) | 0.351 |
|  | Low (< 75 cells/uL) | 91.2% (82.1–100%) |  | 79.4% (66.9–94.2%) |  |
| CD8^+^CD28^+^ T cells | High (≥ 140 cells/uL) | 88.2% (78.0–99.8%) | 0.700 | 61.8% (47.4–80.5%) | 0.013 |
|  | Low (< 140 cells/uL) | 91.2% (82.1–100%) |  | 88.2% (78.0–99.8%) |  |
| CD4^+^CD25^+^ T cells | High (≥ 7 cells/uL) | 82.4% (70.5–96.2%) | 0.045 | 67.6% (53.6–85.3%) | 0.108 |
|  | Low (< 7 cells/uL) | 97.1% (91.5–100%) |  | 82.4% (70.5–96.2%) |  |

*Abbreviations: CI, confidence interval; FFS, failure-free survival; OS, overall survival.*

**Table S6**. Multivariate analysis of risk factors for FFS.

| **Risk factors** | | **HR** | **95% CI** | **P value** |
| --- | --- | --- | --- | --- |
| Age | Per 1-year increase | 0.98 | 0.94-1.02 | 0.289 |
| Conditioning regimen | Bu-included vs. Non-Bu-included | 0.34 | 0.07-1.50 | 0.153 |
| CD8^+^CD28^+^ T cells at 3 months | ≥ 140 vs. < 140 cells/uL | 0.30 | 0.10-0.92 | 0.035 |

*Abbreviations: CI, confidence interval; HR, hazard ratio.*
